# Bilateral adrenal infarction and insufficiency associated with antiphospholipid syndrome and surgery: a case report

**DOI:** 10.1186/s12245-023-00575-0

**Published:** 2023-12-21

**Authors:** Yoshihito Iijima, Masahito Ishikawa, Nozomu Motono, Hidetaka Uramoto

**Affiliations:** Department of Thoracic Surgery, Kanazawa Medical University, 1-1 Daigaku, Uchinada-Machi, Kahoku-Gun, Ishikawa, 920-0293 Japan

**Keywords:** Traumatic hemothorax, Antiphospholipid syndrome, Adrenal insufficiency, Adrenal infarction, Magnetic resonance imaging

## Abstract

**Background:**

Antiphospholipid syndrome causes systemic arterial and venous thromboses due to the presence of antiphospholipid antibodies. Adrenal insufficiency is a rare complication of antiphospholipid syndrome that may result in fatal outcomes if left untreated. Therefore, we report adrenal insufficiency as a rare complication of bilateral adrenal infarction associated with antiphospholipid syndrome and trauma surgery.

**Case presentation:**

A 64-year-old male patient underwent surgery for a left traumatic hemothorax. He concurrently had antiphospholipid syndrome and was receiving warfarin. Postoperatively, the patient complained of severe lumbar back pain despite resuming anticoagulation therapy, and he experienced paralytic ileus and shock. Abdominal contrast-enhanced computed tomography revealed adrenal swelling and increased surrounding retroperitoneal adipose tissue density. Diffusion-weighted abdominal magnetic resonance imaging showed high-intensity areas in the bilateral adrenal glands. Cortisol and adrenocorticotropic hormone levels were 3.30 μg/dL and 185.1 pg/dL, respectively. Subsequently, the patient was diagnosed with bilateral adrenal infarction and acute adrenal insufficiency, and hydrocortisone was immediately administered. Adrenal insufficiency improved gradually, and the patient was discharged after initiating steroid replacement therapy.

**Conclusions:**

The timing of postoperative anticoagulant therapy initiation remains controversial. Therefore, adrenal insufficiency due to adrenal infarction should be monitored while anticoagulant therapy is discontinued in patients with antiphospholipid syndrome.

## Background

Antiphospholipid syndrome (APS) is an autoimmune disease that causes systemic arterial and venous thromboses due to antiphospholipid antibodies (aPL). Adrenal insufficiency (AI) is a rare complication of APS that occurs in only 0.4% of patients [1] and can lead to fatal outcomes if left untreated. The mortality rate of APS complicated with AI is 3.81% [1]. Here, we report AI as a rare complication of bilateral adrenal infarction associated with APS and trauma surgery.

## Case presentation

**Fig. 1:**
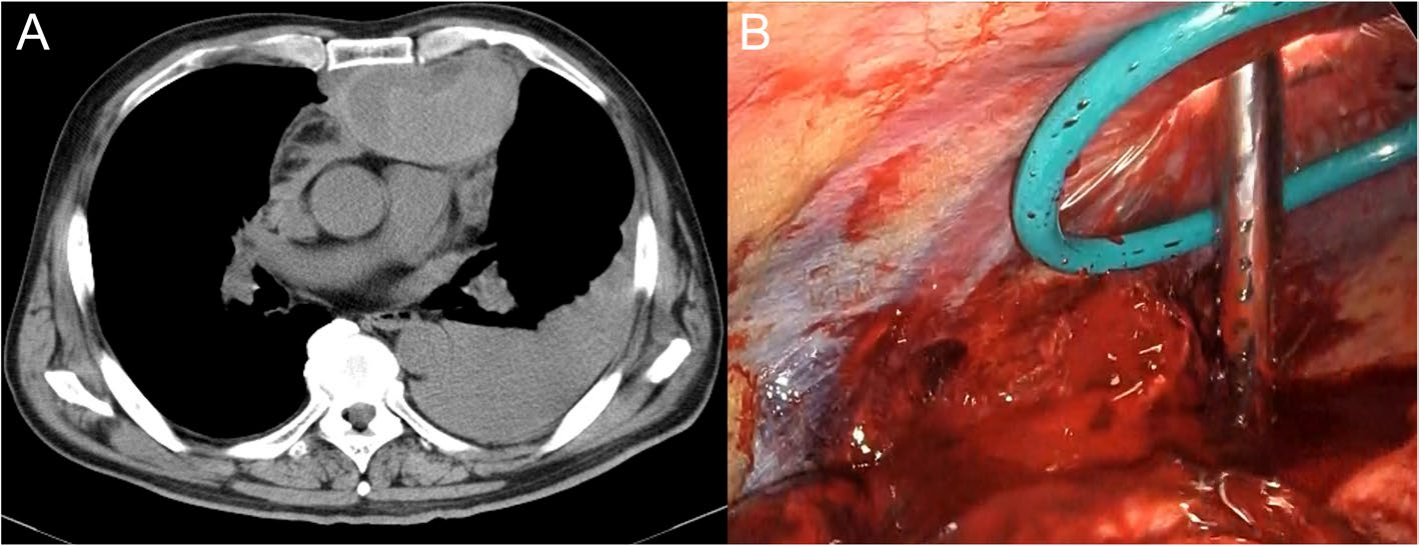
Preoperative and intraoperative findings. **A** Preoperative computed tomography shows a hematoma in the anterior mediastinum and fluid accumulation in the left thoracic cavity. **B** Bleeding from a right internal thoracic vein branch was suspected; however, the bleeding point was unclear

**Fig. 2:**
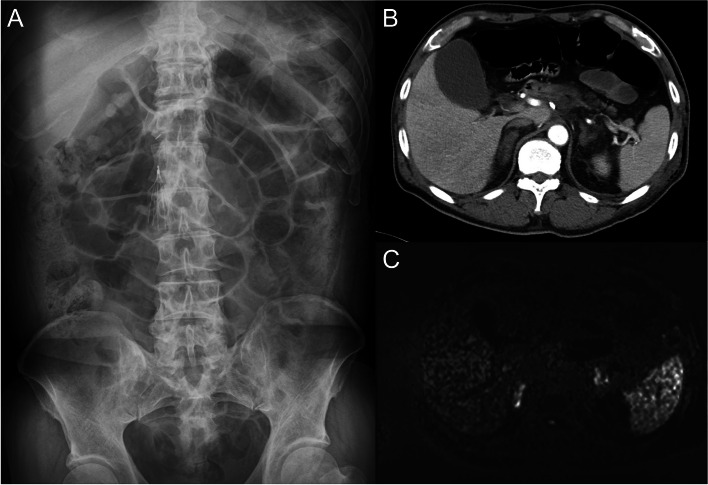
Postoperative findings. **A** Abdominal radiography shows intestinal gas. **B** Contrast-enhanced computed tomography reveals adrenal swelling and increased surrounding retroperitoneal adipose tissue density. **C** Abdominal magnetic resonance imaging reveals high-intensity areas in both adrenal glands on diffusion-weighted images

Ethics approval was not needed for this case report. Written informed consent was obtained from the patient for the publication of this report and its accompanying images. A 64-year-old male patient underwent surgery for a left traumatic hemothorax (Fig. 1A). He concurrently had atrial fibrillation and APS with an inferior vena cava filter placement and was receiving warfarin. The preoperative prothrombin international normalized ratio and hemoglobin level were 3.18 and 5.9 g/dL, respectively, and the blood volume in the thoracic cavity at thoracotomy was 1800 g. Bleeding from a right internal thoracic vein branch was suspected, although the bleeding point was unclear (Fig. 1B). Bleeding could not be controlled with cauterization but was managed using multiple hemostatic agents. The intraoperative blood loss was 200 g; hence, 560 and 480 mL of red blood cells and fresh frozen plasma, respectively, were transfused. Autologous blood (1021 mL) was transfused intraoperatively using a cell saver system. A hematoma extending from the right inguinal to the femoral lesions was observed, and it expanded postoperatively; therefore, the initiation of oral warfarin at 1 mg as an anticoagulation therapy was delayed and resumed on postoperative day (POD) 4. Additional continuous intravenous heparin was administered at 100 IU/kg, and oral warfarin was increased to 2 mg on POD 5. However, the patient complained of severe lumbar back pain on POD 6. Abdominal contrast-enhanced computed tomography (CECT) showed no aortic dissection, intra-abdominal hemorrhage, organ infarction, or adrenal swelling. On POD 8, the patient developed paralytic ileus (Fig. 2A) and shock, and his blood pressure decreased to 70/40 mmHg. Septic shock was ruled out based on blood and urine cultures, and disseminated intravascular coagulation was excluded based on the results of the coagulation fibrinolysis tests. Repeated abdominal CECT revealed adrenal swelling and increased surrounding retroperitoneal adipose tissue density (Fig. 2B). The white blood cell count, C-reactive protein level, sodium level, potassium level, serum creatinine level, glucose level, cortisol level, and adrenocorticotropic hormone level were 8340/mm^3^, 27.54 mg/dL, 130 mmol/L, 4.4 mmol/L, 1.08 mg/dL, 69 mg/dL, 3.30 μg/dL, and 185.1 pg/dL, respectively. Acute AI was suspected, and hydrocortisone (HC) was intravenously administered immediately. Abdominal magnetic resonance imaging revealed high-intensity areas in both adrenal glands on diffusion-weighted images (Fig. 2C), and a diagnosis of AI caused by bilateral adrenal infarctions was made. HC was intravenously administered for 5 days, followed by oral administration at a dosage of 50 mg/day, and AI improved gradually. The patient was discharged on POD 20 after steroid replacement therapy was initiated. HC was tapered and switched to prednisolone (PSL; 5 mg/day) on POD 111. One year has passed since the surgery, and the patient continues to receive PSL (5 mg/day) as an outpatient.

## Discussion and conclusions

APS can cause hemorrhagic adrenal gland infarction and is typically associated with stress factors, including surgery [2]. Bleeding is the leading cause of AI in APS, followed by infarction [1]. Adrenal involvement is usually bilateral [3], and the vascular system of the adrenal gland is unique and complex [3]. Capillary branches from three main arteries form a vascular plexus around the zona reticularis. Furthermore, the transition from the artery to the capillary plexus is known as the “vascular dam,” which is drained by the medullary sinusoids and eventually forms a single central adrenal vein. Venous stasis and hypercoagulability in this region can promote thrombosis, infarction, and subsequent bleeding [3]. Berneis et al. reported a novel mechanism involving the zona fasciculata’s cellular characteristics [4]. In this zone, cells have a high density of late endosomes, which are organelles involved in cholesterol exchange and protein sorting. The membranes of these organelles contain lysobisphosphatidic acid, which is the target of aPLs. These aPLs react locally and promote cholesterol accumulation in cells, leading to cell death and release of lysosomal proteinases, consequently activating endothelial cells, which favors coagulation and causes micro-thromboses [4].

The timing of postoperative anticoagulant therapy initiation is controversial. APS increases the risk of developing venous thromboembolism according to the American College of Chest Physicians guidelines for the perioperative management of antithrombotic therapy [5]. Therefore, heparin bridging is recommended when warfarin is discontinued. However, caution is required when using high-intensity therapeutic dose heparin bridging postoperatively, paticularly in patients undergoing high bleeding-risk surgeries and procedures. If therapeutic dose bridging is used in patients at a high risk of postoperative bleeding, its initiation should be delayed for 48–72 h postoperatively when adequate surgical hemostasis is achieved [5]. However, the intraoperative bleeding point was unclear in our case, and various hematomas were observed from the right inguinal to the femoral lesions. Therefore, heparin and warfarin were resumed 72 h postoperatively. AI caused by adrenal infarction should be monitored while anticoagulant therapy is discontinued in patients with APS.

## Data Availability

All data generated or analyzed during this study are included in this published article.

## Abbreviations

AI: Adrenal insufficiency
aPL: Antiphospholipid antibodies
APS: Antiphospholipid syndrome
CECT: Contrast-enhanced computed tomography
POD: Postoperative day
